# Economic burden and its associated factors of hospitalized patients infected with A (H7N9) virus: a retrospective study in Eastern China, 2013–2014

**DOI:** 10.1186/s40249-016-0170-5

**Published:** 2016-09-01

**Authors:** Xiang Huo, Li-Ling Chen, Lei Hong, Lun-Hui Xiang, Fen-Yang Tang, Shan-Hui Chen, Qiang Gao, Cong Chen, Qi-gang Dai, Chuan-Wu Sun, Ke Xu, Wen-Jun Dai, Xian Qi, Chang-Cheng Li, Hui-Yan Yu, Yin Zhou, Hao-Di Huang, Xing-Yang Pan, Chang-sha Xu, Ming-Hao Zhou, Chang-Jun Bao

**Affiliations:** 1 Department of Acute Infectious Disease, Jiangsu Provincial Center for Disease Control and Prevention, 172 Jiang-su Rd, Nanjing, 210009 China; 2 Suzhou Center for Disease Prevention and Control, Suzhou, China; 3 Nanjing Municipal Center for Disease Control and Prevention, Nanjing, China; 4 Baoshan District Center for Disease Control and Prevention, Shanghai, China; 5 Wuxi Center for Disease Control and Prevention, Wuxi, China; 6 Huaian Center for Disease Control and Prevention, Huaian, China; 7 Changzhou Center for Disease Control and Prevention, Changzhou, China; 8 Xuzhou Center for Disease Control and Prevention, Xuzhou, China; 9 Taizhou Center for Disease Control and Prevention, Taizhou, China; 10 Yancheng Center for Disease Control and Prevention, Yancheng, China; 11 Zhenjiang Center for Disease Prevention and Control, Zhenjiang, China; 12 Yangzhou Center for Disease Control and Prevention, Yangzhou, China; 13 Suqian Municipal Center for Disease Control and Prevention, Suqian, China

**Keywords:** H7N9, Avian influenza, Human infections, Direct medical costs, Hospitalization

## Abstract

**Background:**

H7N9 continues to cause human infections and remains a pandemic concern. Understanding the economic impacts of this novel disease is important for making decisions on health resource allocation, including infectious disease prevention and control investment. However, there are limited data on such impacts.

**Methods:**

Hospitalized laboratory-confirmed H7N9 patients or their families in Jiangsu Province of China were interviewed. Patients’ direct medical costs of hospitalization were derived from their hospital bills. A generalized linear model was employed to estimate the mean direct medical costs of patients with different characteristics.

**Results:**

The mean direct cost of hospitalization for H7N9 was estimated to be ¥ 71 060 (95 % CI, 48 180–104 820), i.e., US$ 10 996 (95 % CI, 7 455–16 220), and was ¥12 060 (US$ 1 861), ¥136 120 (US$ 21 001) and ¥218 610 (US$ 33 728) for those who had mild or severe symptoms or who died, respectively. The principal components of the total fees differed among patients with different disease severity, although medication fees were always the largest contributors. Disease severity, proportion of reimbursement and family member monthly average income were identified as the key factors that contributed to a patient’s direct medical cost of hospitalization.

**Conclusions:**

The direct medical costs of hospitalized patients with H7N9 are significant, and far surpass the annual per capita income of Jiangsu Province, China. The influencing factors identified should be taken into account when developing related health insurance policies and making health resource allocation.

**Trial registration:**

Not applicable. This is a survey study with no health care intervention implemented on human participants.

**Electronic supplementary material:**

The online version of this article (doi:10.1186/s40249-016-0170-5) contains supplementary material, which is available to authorized users.

## Multilingual abstracts

Please see Additional file 1 for translations of the abstract into the six official working languages of the United Nations.

## Background

A novel avian-origin influenza A (H7N9) virus has caused severe human infections in China since February 2013 [1]. As of November 13 2015, a total of 681 laboratory-confirmed cases of human infection with H7N9, including at least 275 deaths, have been reported to World Health Organization (WHO) [2]. Imported cases have been reported in Hong Kong [3], Taiwan [4], Canada [5] and Malaysia [6]. Limited human-to-human transmissions were observed and raised a pandemic concern [7]. Most of the reported human infections have been severe, with 76.6 % admitted to an intensive care unit (ICU), and 27.0 % dying [1]. Over 60 % of cases had underlying medical conditions [8]. These severe symptoms are associated with high direct medical costs. Though understanding the economic burden of H7N9 disease is imperative for understanding the societal and individual impacts of this disease and deciding corresponding health resources allocation, data on this issue are limited. Qi X. et al calculated the direct medical costs for H7N9 patients using formulae, the parameters for which were estimated by experienced hospital financial officers and doctors, from the epidemiological data or from previous publications [9]. In this study, we measured the actual direct medical cost of hospitalized H7N9 patients and its components according to the inventories of patients’ hospitalization fees, and analyzed the influencing factors for the costs. This study describes the actual economic burden of hospitalized patients with H7N9 in order to present evidence on the cost-effectiveness of H7N9 prevention and control.

## Methods

### Subjects

This research was conducted in Jiangsu Province from August to September 2014. All the laboratory-confirmed H7N9 patients who had been hospitalized before the study period were included. Cases were categorized by severity into three groups (mild, severe and fatal). According to the Diagnosis and Treatment Guideline for Human Infections with H7N9 released by the National Health and Family Planning Commission of China (Year 2014), cases with one of the following criteria were defined as having severe infection: a. Leafy lesions or lesions progress >50 % in 48 h indicated by X-ray chest radiograph; b. Dyspnea, breathing rate >24 times/min; c. Severe hypoxemia, patients’ SpO2 ≤ 92 % under oxygen flow of 3–5 litters/min; d. Shock, ARDS (Acute Respiratory Distress Syndrome) or MODS (Multiple Organ Dysfunction Syndrome). Furthermore, patients were grouped into Northern or Southern district based on their street address to study the potential influence of a district’s economic level on medical cost of disease. Generally, both the Per Capita GDP and Per Capita Annual Income are higher in the southern district compared with the northern district of Jiangsu Province, China.

### Information collection

A standardized questionnaire was used to collect information. Trained staff from local centers for disease control and prevention in Jiangsu Province collected data on demographic characteristics, health insurance, proportion of reimbursement and household income through interviewing cases or their family members. Patients’ disease severity were collected via a national system for reporting of notifiable infectious diseases, which were judged and reported by clinical doctors.

### Direct medical costs of hospitalization

Direct medical costs of hospitalized patients infected with H7N9 were derived from patients’ hospital bills. The total cost consists of medication, examination, laboratory tests, treatment, medical consumables, ward bed, nursing and other fees. Examination includes ultrasonography, Computed Tomography (CT), Magnetic Resonance Imaging (MRI), digital imaging and pathological examinations. Laboratory tests include blood, urine, feces and respiratory samples’ testing and culture. Treatment includes diagnosis, monitoring, blood transfusion, oxygen therapy and surgery procedures.

### Statistical analyses

Medians and interquartile ranges (IQRs) were calculated for various medical fees and their accounted proportions. Medical fees were compared between patients with different characteristics, using Mann–Whitney U tests (for 2 groups) or Kruskal-wallis H tests (for multiple groups). All study variables with *P* < 0.2 in univariate analyses were included in multivariate analyses in order to investigate as many potential variables as possible. A generalized linear model (Gamma with log link) was employed to analyze the association between selected variables and patients’ direct medical costs. Both main effects and interactions of selected variables were analyzed. Model’s goodness of fit was compared using Deviance and Chi-Square (per degree of freedom) to choose the best model. Then the selected best model was used to estimate the grand mean of direct medical costs of hospitalized patients infected with H7N9 avian influenza, and that of patients with different characteristics.

## Results

**Fig. 1:**
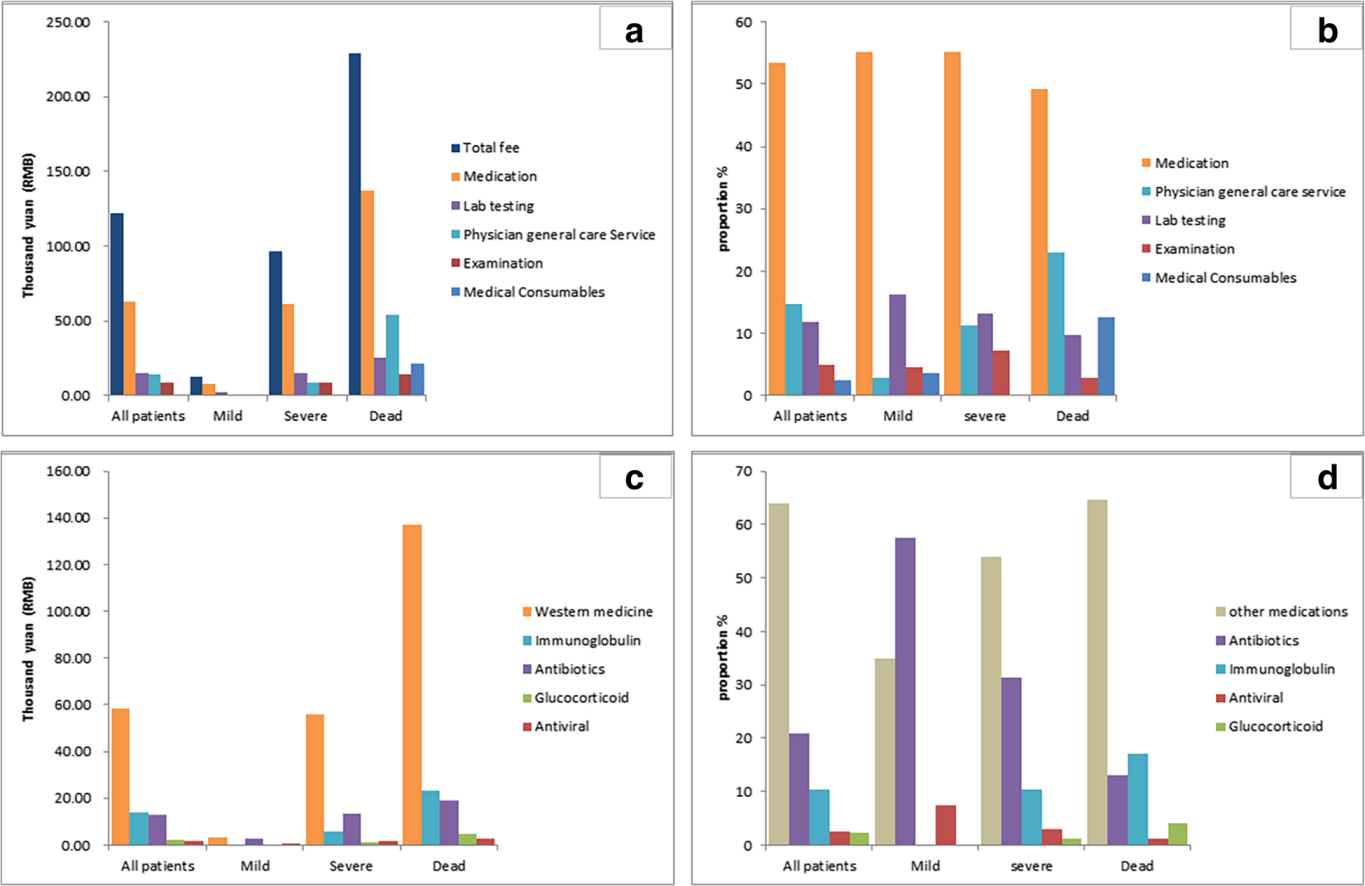
Direct medical costs (thousand yuan, RMB) and proportions of main components for different hospitalized H7N9 patients. **a** Direct medical costs of hospitalization and its main components in different patients; **b** Proportions of main medical cost components in different patients; **c** Medications costs in different patients; **d** Proportions of medications in different patients

Up to 31 July 2014, a total of 52 laboratory-confirmed H7N9 patients were admitted to hospitals and were reported in Jiangsu Province. Twenty-one of them were reported in 2013 and the others in 2014. All of them were included in this study. Male patients accounted for 76.9 %. The median age was 54.5 years (IQR, 36.25–67.50). Among the patients, 4 cases were mild, 27 cases were severe and 21 cases died. The observed median hospitalization fee of all patients studied was ¥ 122 260 (IQR, 60 710–310 220), i.e., US$ 18 858, (IQR, 9 364-47 851; exchange rate, 6.48). The hospitalization fee increased with patient’s disease severity. The median was ¥ 12 790, ¥ 96 780 and ¥ 228 650 for mild, severe and dead patients respectively (*P* < 0.0001). In addition, medication fees, laboratory testing fees, treatment fees and examination fees all increased with patient’s disease severity (*P* < 0.05) (Fig. 1a, Table 1).

**Table 1:** Direct medical costs (RMB, thousand Yuan) of hospitalization in different H7N9 patients

	All patients (*n* = 52)		Mild (*n* = 4)		Severe (*n* = 27)		Dead (*n* = 21)		*P* ^*^	*P* ^**^
	Median (IQR)	Proportion, %, median (IQR)	Median (IQR)	Proportion, %, median (IQR)	Median (IQR)	Proportion, %, median (IQR)	Median (IQR)	Proportion, %, median (IQR)		
Total fee	122 260 (60 710–310 220)	100	12 790 (3 320–19 750)	100	96 780 (56 780–287 140)	100	228 650 (116 410–549 520)	100	<0.0001	--
Examination	8 420 (2 920–17 320)	5.0 (2.5–10.6)	530 (150–1 510)	4.6 (3.5–9.4)	8 660 (3 840–14 700)	7.3 (3.2–11.6)	13 900 (3 400–21 540)	2.8 (1.6–9.9)	0.011	0.092
Lab testing	15 350 (8 520–35 570)	11.9 (9.1–14.9)	1 910 (290–3 390)	16.3 (7.8–19.4)	14 600 (8 470–27 950)	13.2 (10.2–15.3)	25 530 (11 120–65 980)	9.7 (7.6–11.5)	0.002	0.009
Medication	63 250 (27 420–212 700)	53.5 (40.1–60.1)	8 080 (1 820–10 170)	55.1 (34.8–65.3)	61 130 (21 280–151 040)	55.2 (47.0–59.8)	137 410 (54 290–345 040)	49.3 (38.0–60.6)	0.001	0.868
Western medication	58 390 (22 050–190 890)	99.7 (90.6–100.0)	3 520 (780–8 890)	83.6 (36.0–95.9)	55 750 (20 950–146 220)	99.7 (94.6–100.0)	137 220 (54 290–345 030)	99.7 (91.1–100.0)	0.001	0.420
TCM^a^	340 (10–2 490)	0.3(0 .0–3.4)	300 (20–1 700)	12.5 (2.2–19.5)	330 (10–2 420)	0.3 (0.0–3.3)	350 (10–4 870)	0.3 (0.0–2.5)	0.812	0.358
Antibiotics	12 880 (5 370–38 210)	20.8 (11.1–36.3)	2 740 (530–4 950)	57.6 (22.4–68.3)	13 260 (5 870–39 200)	31.4 (16.1–39.0)	19 060 (7 650–43 730)	13.0 (8.1–30.5)	0.014	0.018
Antiviral	1 930 (840–4 130)	2.5 (0.9–7.8)	390 (20–790)	7.5 (1.7–14.2)	1 820 (980–4 140)	2.9 (1.1–8.0)	2 990 (1 390–4 780)	1.2 (0.6–5.7)	0.016	0.333
Glucocorticoids	2 320 (340–8 560)	2.3 (0.4–13.3)	0 (0–1 930)	0.0 (0.0–19.0)	1 140 (270–6 700)	1.3 (0.3–13.6)	4 660 (1 620–22 830)	4.1 (1.6–13.0)	0.002	0.072
Immunoglobulin	13 880 (0–33 400)	10.4 (0.0–24.4)	0	0.0	5 960 (0–39 100)	10.4 (0.0–31.6)	23 040 (8 290–39 780)	17.0 (9.4–21.5)	0.014	0.025
Treating	13 860 (5 610–65 210)	14.8 (8.3–23.8)	120 (70–2 500)	2.9 (0.8–12.9)	8 770 (4 600–32 350)	11.2 (7.7–18.3)	54 450 (16 170–200 570)	22.9 (14.8–29.8)	<0.0001	0.002
Medical Consumables	1 010 (0–16 510)	2.4 (0.0–12.2)	300 (100–2 290)	3.7 (2.2–11.8)	150 (0–6 070)	0.2 (0.0–5.9)	21 630 (0–88 810)	12.6 (0.0–19.1)	0.025	0.020
Sick bed	2 400 (1 340–3 990)	1.4 (0.7–3.2)	320 (1 650–5 260)	4.4 (1.1–14.2)	3 040 (1 430–5 150)	2.1 (1.2–4.3)	2 240 (1 410–3 520)	0.8 (0.5–1.4)	0.021	0.001
Nursing	2 390(790–6 270)	1.5 (0.7–3.3)	340 (160–660)	5.3 (0.9–18.9)	3 170 (1 250–6 620)	2.6 (1.1–4.8)	2 180 (1 490–7 850)	1.3 (0.7–1.5)	0.050	0.033
Other fees	100 (0–810)	0.1 (0.0–1.1)	10 (0–70)	0.1 (0.0–0.3)	310 (70–2 400)	0.4 (0.1–2.0)	60 (0–260)	0.0 (0.0–0.2)	0.022	0.032

^*^P for fee medians comparisons among patients with different clinical outcomes, using Kruskal-wallis H test

^**^P for proportion medians comparisons among patients with different clinical outcomes, using Kruskal-wallis H test

^a^Traditional Chinese Medicines

Medication costs accounted for the largest portion of the hospitalization fees (median proportion, 53.5 %), followed by treatment costs (median proportion, 14.8 %) and laboratory testing costs (median proportion, 11.9 %). The principal components of the hospitalization fees differed among patients with different disease severity, although medication fees were always the highest proportion (55.1, 55.2 and 49.3 % for mild, severe and dead patients respectively, *P* = 0.868). The medication cost was followed by laboratory testing cost (16.3 %) and examination cost (4.6 %) in mild patients, by laboratory testing cost (13.2 %) and treatment cost (11.2 %) in severe patients and by treatment cost (22.9 %) and medical consumables cost (12.6 %) in dead patients. The proportion of laboratory testing costs and examination costs declined, while the proportion of treatment costs and medical consumables cost increased with patient’s disease severity (*P* < 0.05) (Fig. 1b, Table 1).

Most of the medication used was western medication (median proportion, 99.7 %), with no significant difference among patients with different disease severity (*P* = 0.420). The median western medication costs were ¥ 3 520, ¥55 750 and ¥137 220 for mild, severe and dead patients (*P* = 0.001). Antibiotics, antivirals, glucocorticoids and immunoglobulin costs all increased with patients’ disease severity (*P* < 0.05) (Fig. 1c, Table 1).

In mild patients, antibiotic expenses accounted for over a half (proportion median, 57.6 %) of the medication fee and antivirals expense accounted for 7.5 %. These proportions declined with patients’ disease severity. Proportion of antibiotics expense decreased to 31.4 and 13 % for severe and dead patients, and antivirals expense decreased to 2.9 and 1.2 % respectively. In contrast, the proportion of immunoglobulin and glucocorticoids expense increased with patient’s disease severity. Other drugs expense increased with patient’s disease severity as well (Fig. 1d, Table 1).

Stratified analyses indicated that significant difference in hospitalization fee was not found among patients with different types of health insurances (*P* = 0.755), but was found among patients with different proportions of reimbursement (*P* = 0.033). Hospitalization fees were significantly higher in patients from the southern district of Jiangsu Province (*P* = 0.044) and obviously lower in patients with a family member monthly average income of less than ¥ 1 000 (*P* = 0.135). A significant higher hospitalization fee was observed in female patients (*P* = 0.020) compared with male patients. Nevertheless, no significant difference was observed among patients of different ages ((*P* = 0.945) (Table 2).

**Table 2:** Univariate analysis of the factors associated with direct medical cost (RMB) of hospitalized H7N9 patients

Selected variables	Frequency	Median of total fee (interquartile range)	***P*** ^*^
Overall	52	122 260 (60 710–310 220)	N.A.
Severity and outcome			<0.0001
Mild	4	12 780 (3 320–19 750)	
Severe	27	96 780 (56 780–287 140)	
Dead	21	228 650 (116 410–549 520)	
Gender			0.020
Male	40	100 110 (52 190–265 010)	
Female	12	259 880 (116 410–544 590)	
Age, years			0.945
<34	10	201 960 (21 750–460 340)	
35–64	27	108 420 (64 350–277 420)	
>=65	15	123 820 (80 350–310 210)	
District^a^			0.044
Northern	12	84 840 (37 230–125 650)	
Southern	39	138 360 (76 740–460 660)	
Health insurance^a^			0.755
None	5	96 780 (57 360–287 140)	
URBMI	28	92 210 (52 190–271 140)	
NCMS	12	237 550 (62 130–291 280)	
Proportion of Reimbursement			0.033
<50 %	7	96 780 (38 800–128 980)	
50–79 %	10	101 140 (53 990–228 650)	
80–99 %	8	87 270 (29 700–193 050)	
100 %	27	254 340 (93 510–549 520)	
Family member monthly average income (RMB, thousand yuan)^a^			0.135
<1	4	67 770 (37 870–167 950)	
1–3	10	303 340 (120 700–606 530)	
3–5	15	105 460 (77 960–165 360)	
5–10	14	131 640 (32 330–460 340)	
>=10	6	302 140 (123 820–1103 550)	

^*^Mann–Whitney *U* test (for 2 groups) or Kruskal-wallis H test (for multiple groups)

^a^There are 1, 7 and 3 missing data in District, Health insurance and Family member monthly average income respectively

All variables with *P* < 0.2 aforementioned were included in the multivariate analyses using a generalized linear model (Gamma with log link). Finally, the combination of disease severity, proportion of reimbursement and family member monthly average income was found to be associated with patients hospitalization fee with best goodness of fit (Additional file 2: Table S1). The fitted model estimated that the mean direct medical costs of hospitalized patients infected with H7N9 was ¥71 060 (95 % *CI*, 48 180–104 820), which was 2.18 times the annual per capita disposable income of urban residents, and 5.23 times the annual per capita net income of rural residents of Jiangsu Province, China in 2013 [10]. As of Nov. 13 2015, a total of 78 human infections with H7N9 were confirmed and 74 of them were hospitalized in Jiangsu Province. Thus, the total direct medical costs of hospitalized H7N9 patients in Jiangsu Province were estimated to be ¥ 5 258 440 (95 % *CI*, 3 565 320–7 756 680), i.e., US$ 811 594 (95 % *CI*, 550 276–1 197 176).

The mean hospitalization fees of patients with different disease severity were also estimated by the fitted model. The means were estimated to be ¥12 060, ¥136 120 and ¥218 610 for mild, severe and dead patients, respectively. In addition, mean hospitalization fees of patients with different proportions of reimbursement and family member monthly average income were estimated by the fitted model as well. Hospitalization fees were significantly higher in patients with 100 % reimbursement and significantly lower in patients with family member monthly average income lower than ¥ 1 000 compared with the mean fees for all patients (*P* = 0.026 and 0.002) (Table 3).

**Table 3:** Estimated direct medical cost (RMB) of hospitalization for H7N9 patients with different characteristics

Selected variables	B	*P* ^*^	Estimated Mean (95 % *CI*)	*P* ^**^
Severity				
Mild	Ref.	N.A.	12 060 (4 710–30 860)	<0.0001
Severe	2.424	<0.0001	136 120 (88 830–208 580)	0.557
Dead	2.897	<0.0001	218 610(134 200–356 100)	0.013
Reimbursement proportion				
<50 %	Ref.	N.A.	46 010 (21 480–98 570)	0.276
50–79 %	0.340	0.526	64 670 (32 060–130 470)	0.415
80–99 %	0.233	0.667	58 060 (27 900–120 840)	0.415
100 %	1.166	0.012	147 630 (92 610–235 340)	0.026
Family member monthly average income (RMB, thousand yuan)				
<1	Ref.	N.A.	22 250 (9 150–54 130)	0.002
1–3	1.691	0.001	120 770 (64 890–224 770)	0.613
3–5	1.236	0.012	76 600 (47 320–123 990)	0.730
5–10	1.124	0.020	68 460 (41 430-113 110)	0.662
>=10	1.755	0.002	128 620 (56 990-290 320)	0.613

Dependent Variable: Total fee

Model: (Intercept), severity, Reimbursement proportion, Family member monthly average income

Grand mean (95 % CI): 71 060 (48 180-104 820)

^*^P value for coefficient B

^**^P value for estimated mean compared with grand mean

## Discussion

This study measured the direct medical costs associated with hospitalization with H7N9 avian influenza in Jiangsu Province, China. The direct medical costs were found to be significant, far surpassing the annual per capita income for residents of Jiangsu Province. This disparity in costs and income was especially high in those who died from the infection. The total economic burden would be even higher if patients’ direct medical costs before admission, direct non-medical expenses and indirect economic losses due to deaths or missed working time were included [9]. The mean direct cost of hospitalization with H7N9 (US$ 10 969) estimated in this research is much higher than that of hospitalization associated with seasonal influenza (US$ 1 797) [11] and the healthcare costs per patient with severe acute respiratory syndrome (SARS) in Beijing (US$ 1 886) most likely due to higher rates of ICU admission and death [12, 13] and the use of more expensive modern medical devices [14]. In addition, the longer hospitalization duration of H7N9 patients (compared with H5N1 and pH1N1, *P* < 0.001) could also be responsible for the higher hospitalization fee [15]. According to the mean cost (US$ 10 969; 95 % *CI*, 7 356–16 003) determined in this study, we estimate that the total direct medical costs associated with hospitalization of H7N9 patients across China as of November 13, 2015 (681 patients reported, assuming the hospitalization rate is 92.9 % which is the rate found in Jiangsu Province) is about US$ 6 932 408 (95 % *CI*, 4 648 992–10 113 896).

Local governments provided additional financial support for patients infected with H7N9 at the beginning of the H7N9 epidemic. In this study, over a half (51.9 %) of the patients got full reimbursement of their hospitalization fees, and about 67 and 87 % of the patients got at least 80 and 50 % of their hospitalization fees reimbursed. This alleviated the actual economic burden of H7N9 patients and their families greatly. However, this additional financial support was not continued. We found in our study that hospitalization fees of patients with family member monthly average income lower than ¥ 1 000 was significantly lower than the average even after adjusting for disease severity and reimbursement proportion. This is because, for the low income group, even though the health insurance can provide some financial protection, the co-payment required is still too high for many to afford [16]. Thus, policies and regulations should be made to provide more financial support for low-income groups who contract H7N9 and other similar severe infectious diseases. In addition, patients with full reimbursement were found to have a significantly higher hospitalization fee compared with the average after adjusting for disease severity and family member average income. This should also be taken into account when making related policies and regulations in order to avoid unnecessary medical costs, as it has been reported that reimbursement restrictions had a positive impact on enhancing the efficiency of antihypertensive prescribing [17].

The mean direct medical costs of hospitalized H7N9 patients were found to be ¥12 060 (US$1 861), ¥136 120 (US$21 006) and ¥218 610 (US$33 736) (estimated by model), or ¥12 790 (US$1 974), ¥96 780 (US$14 935) and ¥228 650 (US$35 285) (medians observed) for mild, severe and dead cases respectively in this study. Qi X. et al indirectly calculated the direct medical costs of H7N9 patients in China using parameters estimated by experienced hospital financial officers and doctors. Their results show that the mean cost for each patient was ¥10 117 (US$ 1 619) for mild patients, ¥139 323 (US$22 292) for severe cases without death and ¥205 976 (US$32 956) for severe cases with death [9], which are comparable with our data particularly as it should be noted that the direct medical costs calculated by Qi X et al were the total costs across a patients’ whole disease course, while the costs we investigated here were only hospitalization fees. Thus, it is reasonable to infer that the cost of hospitalization accounts for an overwhelming majority of a patient’s total medical cost. In addition, the difference in medical cost between Jiangsu Province and other districts of China should not be overlooked. For example, New Cooperative Medical Scheme, one of the major types of health insurance in China, has provincial differences in funding [16]. This difference could further lead to provincial differences in medical cost, as the reimbursement rate is found to influence the direct medical cost of hospitalization in this study.

Medication, treatment and laboratory testing expenses comprised the major part (80.2 %) of direct medical cost of hospitalization with H7N9; a finding similar with that of seasonal influenza-related hospitalizations, where therapeutics and diagnostics were the two largest components of direct medical cost, comprising 57 and 23 %, respectively [11].

The molecular mechanisms of traditional Chinese medicine (TCM) for the treatment of influenza virus infection, including H7N9, have been indicated by computational approaches [18]. A preliminary study suggested that duration of TCM therapy might has an inverse association with patient’s critical outcome [19]. The Diagnosis and Treatment Guideline for Human Infections with H7N9 released by National Health and Family Planning Commission of China also recommend TCM therapy. However, TCM expense was found to be quite low in this studied patients group. Further studies should be conducted to establish the evidence on the effectiveness of TCM therapy, further facilitating TCM administration.

We found that other drugs expense (other than antibiotics, antivirals, glucocorticoids and Immunoglobulin) accounted for a large proportion (over a half) of hospitalization fees for severe or dead patients. The proportion of immunoglobulin expense was much lower (no more than 17 %). In the study of Qi X. et al, other drugs expense was estimated to be ¥270 per day for mild patients and ¥1 600 per day for severe patients, which was consistent with our findings. However, they estimated the expense of immunoglobulin to be ¥2 500 per day, which is seriously overestimated according to our observed data [9].

The expense of glucocorticoids was found to increase with patients’ disease severity in this study. However, glucocorticoids were found to enhance replication of influenza viruses [20] and a research from Vietnam indicated that no significant effectiveness for survival was observed among H5N1 patients treated with methylprednisolone [21]. Thus, more evidence is needed to guide clinical administration of glucocorticoids.

There are limitations in our study. First, only direct medical cost of hospitalization was described. Knowing the medical expenses before patients’ admission and the indirect economic losses associated with the loss of working time for H7N9 patients and their family members would present a more complete portrayal of the economic burden of this disease [22]. Second, the sample size is small, especially for mild patients, although we have included all mild patients with hospitalization in Jiangsu Province during the study period. The special disease spectrum of H7N9 infection (most are severe) or difficulties in identifying mild patients (no comprehensive administration of H7N9 test in influenza-like-illness outpatients) might be responsible [23].

## Conclusion

In conclusion, this study described the actual direct medical cost of hospitalization with H7N9 and the main components of the cost, helping us better understand the economic burden of this novel disease and better identify the cost-benefits of investing in allocating resources to prevention and control activities. Furthermore, the combination of disease severity, reimbursement proportion and family member monthly average income was found to be associated with patient’s hospitalization fee and these factors should be addressed in making related health insurance policies. Our results also pose some questions in clinical medication administration, which need to be further investigated. The mean hospitalization fees presented by this study could be used for estimating the total direct medical costs of Jiangsu Province and even that of China after adjusting the identified determinants aforementioned.

## Abbreviations

ARDS, Acute Respiratory Distress Syndrome; CDC, Center for Disease Control and Prevention; CT, computed tomography; ICU, intensive care unit; IQR, interquartile range; MODS, Multiple Organ Dysfunction Syndrome; MRI, Magnetic Resonance Imaging; WHO, World Health Organization.

## Additional file

Additional file 1: Multilingual abstracts in the six official working languages of the United Nations. (PDF 283 kb) Additional file 2: Table S1. Generalized linear models (Gamma with log link) selection. (DOCX 30 kb)
