# Supplementary material for: Economic burden and its associated factors of hospitalized patients infected with A (H7N9) virus: a retrospective study in Eastern China, 2013–2014
Source: Infect Dis Poverty. 2016 Sep 1;5(1):79. doi: 10.1186/s40249-016-0170-5 (PMC5007809; doi:10.1186/s40249-016-0170-5)
Supplement: Additional file 1: — Multilingual abstracts in the six official working languages of the United Nations. (PDF 283 kb) [file 40249_2016_170_MOESM1_ESM.pdf]

Translation of the abstract into the six official working languages of the United Nations

العبء الاقتصادي والعوامل المرتبطة به للمرضى في المستشفى المصابين بفيروس (A (H7N9: دراسة استعادية في شرق الصين، 2014-2013

شيانج هوه، لي- لينج تشن، لي هونج ، لون- هوي شيانج، فين- يانج تانج، شان- هوي تشن، تشيانج جاو، تسونج تشن، تشي- جانج داي، شوان- وو سن، كه شو، ون- جن داي، شيان تشي، تشانج- تشنج لي، هوى- يان يو، يين تشو، هاو- دي هوانج، شينج- يانج بان، تشانج- شا شو، مينج- هاو تشو، تشانج-جن باو

الملخص

الخلفية: يواصل H7N9 التسبب في إصابات بشرية، ولا يزال مصدر قلق وبائي. ومن المهم فهم الآثار الاقتصادية لهذا المرض الجديد لاتخاذ قرارات بشأن تخصيص الموارد الصحية، بما في ذلك الوقاية من الأمراض المعدية والاستثمار في السيطرة. ومع ذلك، هناك بيانات محدودة عن تلك الآثار.

الطرق: تم مقابلة المرضى المنومين بالمستشفى المؤكد مختبرياً إصابتهم بـ H7N9 أو أسرهم في مقاطعة جيانجسو الصينية. وقد تم استنتاج التكاليف الطبية المباشرة للمرضى المنومين من فواتيرهم بالمستشفى. تم استخدام نموذج خطي معمم لتقدير التكاليف الطبية المتوسطة المباشرة لمرضى يعانون من خصائص مختلفة.

النتائج: قدرت التكلفة المتوسطة المباشرة للتنويم بالمستشفى بسبب H7N9 لتكون 71060 يوان (95٪ فاصل ثقة، 820 180-104 48)، أي 10996 دولار أمريكي (95٪ فاصل ثقة، 220 455-16 7)، وكان 12060 يوان (1861 دولار أمريكي)، 136120 يوان (21001 دولار أمريكي) و 218610 يوان (33728 دولار أمريكي) بالنسبة لأولئك الذين لديهم أعراض خفيفة أو شديدة أو الذين لقوا حتفهم، على التوالي. اختلفت المكونات الرئيسية لمجموع الرسوم بين المرضى الذين يعانون من اختلاف شدة المرض، على الرغم من أن رسوم الدواء كانت دائماً أكبر المسهمين. وقد تم تحديد شدة المرض، ونسبة السداد ومتوسط الدخل الشهري لأفراد الأسرة ، كعوامل رئيسية ساهمت في التكاليف الطبية المباشرة للمريض في المستشفى.

الاستنتاجات: إن التكاليف الطبية المباشرة للمرضى بالمستشفى بفيروس H7N9 كبيرة، وتتجاوزحتى الدخل السنوي للفرد في مقاطعة جيانجسو، بالصين. ويجب أن تؤخذ بعين الاعتبار العوامل المؤثرة المحددة عند وضع سياسات التأمين الصحي ذات الصلة، وتقدير حصة الموارد الصحية.

Translated from English version into Arabic by Free bird, through

TRANSLATORS WITHOUT BORDERS

H7N9 禽流感住院病例的经济负担及其影响因素：2013-2014 年中国东部的一项回顾性研究

霍翔，陈立凌，洪镭，向伦辉，汤奋扬，陈善辉，高强，陈聪，戴启刚，孙传武，许可，戴文军，祁贤，李长城，余慧燕，周印，黄昊頔，潘兴扬，许长沙，周明浩，鲍倡俊

摘要

引言：H7N9 禽流感病毒持续造成人类感染并存在大流行的风险。了解这种新发传染病的经济学影响对于卫生资源分配的决策，包括感染性疾病预防和控制的投入，是非常重要的。然而目前这方面的数据非常有限。

方法：对中国江苏省经实验室确诊的 H7N9 住院病例或其家属进行面访调查。根据病例的医院账单获取其住院的直接医疗费用。使用广义线性模型估计不同特征病例的平均直接医疗费用。

结果：据模型推断，H7N9 住院病例的平均直接医疗费用为人民币 71 060 元（95%可信区间：48 180-104 820），即美元 10 996 元（95%可信区间：7 455-16 220），轻症、重症和死亡病例的平均费用分别为人民币 12 060 元（美元 1 861 元）、人民币 136 120 元（美元 21 001 元）和人民币 218 610 元（美元 33 728 元）。不同严

重程度病例的主要费用构成不同，但医药费均占最大比例。疾病严重程度、报销比例和家庭成员平均月收入是影响住院病例直接医疗费用的重要因素。
结论：H7N9 住院病例的直接医疗费用高，远超中国江苏省的年人均收入水平。在制定相关医疗保险政策和进行卫生资源分配时，应重点关注本研究发现的影响直接医疗费用的关键因素。

Translated from English version into Chinese by Hao-Di Huang

Le fardeau économique des patients hospitalisés pour infection par le virus A (H7N9) : étude rétrospective en Chine orientale, 2013-2014

Xiang Huo, Li-Ling Chen, Lei Hong, Lun-Hui Xiang, Fen-Yang Tang, Shan-Hui Chen, Qiang Gao, Cong Chen, Qi-gang Dai, Chuan-Wu Sun, Ke Xu, Wen-Jun Dai, Xian Qi, Chang-Cheng Li, Hui-Yan Yu, Yin Zhou, Hao-Di Huang, Xing-Yang Pan, Chang-sha Xu, Ming-Hao Zhou, Chang-Jun Bao

Résumé
Contexte: Le virus H7N9 continue de causer des infections humaines et il demeure une préoccupation pandémique. Il est important de comprendre les impacts économiques de cette nouvelle maladie pour la prise de décisions sur la répartition des ressources de santé, en ce compris l'investissement pour la prévention et le contrôle des maladies infectieuses. Cependant, il existe peu de données sur ces impacts.
Méthodes: Des patients de la province chinoise du Jiangsu, hospitalisés pour H7N9 confirmé au laboratoire, ou leurs familles ont été interrogés. Les coûts médicaux directs des hospitalisations pour les patients ont été dérivés de leurs factures d'hôpital. Un modèle linéaire généralisé a été utilisé pour estimer les coûts médicaux directs moyens pour des patients présentant diverses caractéristiques.
Résultats: Le coût direct moyen d'une hospitalisation pour H7N9 a été estimé à 71 060 ¥ (IC à 95 % : 48 180-104 820), soit 10 996 $ US (IC 95 % : 7 455-16 220), et il était de 12 060 ¥ (1 861 $ US), 136 120 ¥ (21 001 $ US) et 218 610 ¥ (33 728 $ US) pour ceux qui avaient des symptômes bénins, graves, ou qui sont morts, respectivement. Les principales composantes du total des frais différaient chez les patients selon leur degré de gravité de maladie, mais les coûts des médicaments étaient toujours les plus gros contributeurs. La gravité de la maladie, la proportion du remboursement et le revenu mensuel moyen des membres de la famille ont été identifiés comme les principaux facteurs contribuant au coût médical direct d'hospitalisation d'un patient.
Conclusions: Les coûts médicaux directs des patients hospitalisés pour H7N9 sont importants et ils dépassent de loin le revenu annuel par habitant de la province du Jiangsu, en Chine. Les facteurs d’influence identifiés doivent être pris en compte lors de l'élaboration des politiques d'assurance santé et de la répartition des ressources de santé.

Translated from English version into French by Jacek Sierakowski, through

TRANSLATORS
WITHOUT BORDERS

Экономическое бремя и сопутствующие факторы госпитализации больных, инфицированных вирусом A (H7N9): ретроспективное исследование в Восточном Китае, 2013-2014 года

Xiang Huo, Li-Ling Chen, Lei Hong, Lun-Hui Xiang, Fen-Yang Tang, Shan-Hui Chen, Qiang Gao, Cong Chen, Qi-gang Dai, Chuan-Wu Sun, Ke Xu, Wen-Jun Dai, Xian Qi, Chang-Cheng Li, Hui-Yan Yu, Yin Zhou, Hao-Di

Huang, Xing-Yang Pan, Chang-sha Xu, Ming-Hao Zhou, Chang-Jun Bao

Аннотация

Справочная информация: H7N9 продолжает быть причиной инфицирования людей и остается проблемой пандемии. Понимание экономических последствий этого нового вида заболевания имеет важное значение для принятия решений о распределении ресурсов здравоохранения, в том числе инвестиции в профилактику и контроль инфекционных заболеваний. Однако, имеются лишь ограниченные данные о подобных последствиях.

Методы: Были опрошены госпитализированные больные с лабораторно подтвержденным вирусом H7N9 и их семьи из провинции Цзянсу, Китай. Расходы на госпитализацию больных были рассчитаны на основе их больничных счетов. Обобщенная линейная модель использовалась для оценки средней прямых медицинских расходов пациентов с различными характеристиками.

Результаты: Средняя прямая стоимость госпитализации для H7N9 составила ¥ 71 060 (95% CI, 48 820 180-104), то есть $ 10 996 (95% CI, 7 455-16 220), и было ¥ 12 060 (US$ 1 861), ¥ 136 120 (США $ 21 001) и ¥ 218 610 ($ 33 728) для тех, кто имел легкие или тяжелые симптомы или кто умер, соответственно. Основные факторы расчета общей суммы разнятся среди пациентов в зависимости от степени заболевания, хотя самые большие суммы всегда были за медикаменты. Тяжесть заболевания, доля возмещения и среднемесячный доход членов семьи были ключевыми факторы, которые способствовали расчету прямых медицинских расходов пациента на госпитализацию.

Выводы: Прямые медицинские расходы пациентов, госпитализированных с H7N9, являются значительными и в несколько раз превосходят годовой доход на душу населения китайской провинции Цзянсу. Все факторы влияния должны быть учтены при разработке соответствующей политики медицинского страхования и при распределении ресурсов здравоохранения.

Translated from English version into Russian by Daria Toropchyn, through

TRANSLATORS
WITHOUT BORDERS

Carga económica y factores asociados de los pacientes hospitalizados infectados con el virus A (H7N9): estudio retrospectivo en el este de China 2013-2014

Xiang Huo, Li-Ling Chen, Lei Hong, Lun-Hui Xiang, Fen-Yang Tang, Shan-Hui Chen, Qiang Gao, Cong Chen, Qi-gang Dai, Chuan-Wu Sun, Ke Xu, Wen-Jun Dai, Xian Qi, Chang-Cheng Li, Hui-Yan Yu, Yin Zhou, Hao-Di Huang, Xing-Yang Pan, Chang-sha Xu, Ming-Hao Zhou, Chang-Jun Bao

Resumen

Antecedentes: H7N9 sigue causando infecciones en humanos y sigue siendo una preocupación pandémica. La comprensión de los impactos económicos de esta nueva enfermedad es importante para tomar decisiones sobre la asignación de recursos para la salud, incluyendo la prevención de enfermedades infecciosas y su control. Sin embargo, los datos sobre tales impactos son limitados.

Métodos: Se entrevistó a pacientes hospitalizados con H7N9 confirmado en laboratorio o a sus familias, en la provincia de Jiangsu China. Los costes médicos directos de los pacientes por la hospitalización se derivaron de sus

cuentas del hospital. Se utilizó un modelo lineal generalizado para estimar el promedio de los costes médicos directos de los pacientes con diferentes características.

Resultados: El costo directo promedio de hospitalización por H7N9 se estimó en ¥ 71 060 (95%*IC*, 48 180-104 820), es decir, US$ 10 996 (95%*IC*, 7 455-16 220) y fue ¥ 12 060 (1 US$ 861),
¥ 136 120 (US$ 21 001) y ¥ 218 610 (US$ 33 728) para los que tenían síntomas leves o graves o que murieron, respectivamente. Los principales componentes de los gastos totales diferían entre los pacientes con diferente gravedad de la enfermedad, aunque los costos de los medicamentos fueron en todos los casos, los que contribuyeron más. La gravedad de la enfermedad, el porcentaje del reembolso y el ingreso promedio mensual del miembro de la familia, se identificaron como los factores clave que contribuyeron al costo médico directo por la hospitalización del paciente.

Conclusiones: Los costos médicos directos de los pacientes hospitalizados con H7N9 son significativos y muy superiores a los ingresos anuales per capita en la provincia de Jiangsu, China. Los factores influyentes identificados deben ser tenidos en cuenta en el desarrollo de las pólizas de seguro de salud y en la asignación de recursos para la salud.

Translated from English version into Spanish by Patriciacassoni, through

TRANSLATORS
WITHOUT BORDERS
