# Supplementary material for: Economic burden and its associated factors of hospitalized patients infected with A (H7N9) virus: a retrospective study in Eastern China, 2013–2014
Source: Infect Dis Poverty. 2016 Sep 1;5(1):79. doi: 10.1186/s40249-016-0170-5 (PMC5007809; doi:10.1186/s40249-016-0170-5)
Supplement: Additional file 2: Table S1. — Generalized linear models (Gamma with log link) selection. (DOCX 30 kb) [file 40249_2016_170_MOESM2_ESM.docx]

**Supplement**

**Table S1.** Generalized linear models (Gamma with log link) selection

| Fitted models ^1^ | Variables number | Model description | Omnibus Test ^3^ (P) | Goodness of Fit (Value/df) ^4^ | |
| --- | --- | --- | --- | --- | --- |
|  |  |  |  | Deviance | Pearson Chi-Square |
| **Disease severity**, Gender, District, **Reimbursement proportion**, **FMMAI** ^2^ | 5 | Main effects | < 0.0001 | 0.981 | 0.943 |
| **Disease severity**, **Reimbursement proportion**, **FMMAI** ^2^ | 3 | Main effects | < 0.0001 | 0.974 | 0.893 |
| **Disease severity**, **Reimbursement proportion**, **FMMAI**, **Disease severity * Reimbursement proportion * FMMAI** | 4 | Main effects and 3-way interaction | < 0.0001 | 1.099 | 1.023 |
| **Disease severity**, Reimbursement proportion, FMMAI, Disease severity * Reimbursement proportion, Disease severity * FMMAI, Reimbursement proportion * FMMAI | 6 | Main effects and all 2-way interactions | < 0.0001 | 1.142 | 1.166 |

^#^ Variables in old characters have statistically significant model effects (P < 0.05).

^1^ Intercept is included in models.

^2^ FMMAI, short for Family member monthly average income

^3^ Compares the fitted model against the intercept-only model.

^4^ Information criteria are in smaller-is-better form.
